# Repertoire of *Escherichia coli *agonists sensed by innate immunity receptors of the bovine udder and mammary epithelial cells

**DOI:** 10.1186/1297-9716-43-14

**Published:** 2012-02-13

**Authors:** Adeline Porcherie, Patricia Cunha, Angelina Trotereau, Perrine Roussel, Florence B Gilbert, Pascal Rainard, Pierre Germon

**Affiliations:** 1 INRA, UMR 1282 Infectiologie et Santé Publique, F-37380 Nouzilly, France; 2 Université François Rabelais, UMR 1282, Tours, France

## Abstract

*Escherichia coli *is a frequent cause of clinical mastitis in dairy cows. It has been shown that a prompt response of the mammary gland after *E. coli *entry into the lumen of the gland is required to control the infection, which means that the early detection of bacteria is of prime importance. Yet, apart from lipopolysaccharide (LPS), little is known of the bacterial components which are detected by the mammary innate immune system. We investigated the repertoire of potential bacterial agonists sensed by the udder and bovine mammary epithelial cells (bMEC) during *E. coli *mastitis by using purified or synthetic molecular surrogates of bacterial agonists of identified pattern-recognition receptors (PRRs). The production of CXCL8 and the influx of leucocytes in milk were the readouts of reactivity of stimulated cultured bMEC and challenged udders, respectively. Quantitative PCR revealed that bMEC in culture expressed the nucleotide oligomerization domain receptors NOD1 and NOD2, along with the Toll-like receptors TLR1, TLR2, TLR4, and TLR6, but hardly TLR5. In line with expression data, bMEC proved to react to the cognate agonists C12-iE-DAP (NOD1), Pam3CSK4 (TLR1/2), Pam2CSK4 (TLR2/6), pure LPS (TLR4), but not to flagellin (TLR5). As the udder reactivity to NOD1 and TLR5 agonists has never been reported, we tested whether the mammary gland reacted to intramammary infusion of C12-iE-DAP or flagellin. The udder reacted to C12-iE-DAP, but not to flagellin, in line with the reactivity of bMEC. These results extend our knowledge of the reactivity of the bovine mammary gland to bacterial agonists of the innate immune system, and suggest that *E. coli *can be recognized by several PRRs including NOD1, but unexpectedly not by TLR5. The way the mammary gland senses *E. coli *is likely to shape the innate immune response and finally the outcome of *E. coli *mastitis.

## Introduction

Mastitis is an important pathology in the dairy industry, both in terms of economic impact and animal health. *Escherichia coli *is among the major mastitis pathogens responsible for clinical mastitis in cows [1-4]. The infection is initiated by the entry of the bacteria through the teat canal and, after a short incubation period, is characterized by an important inflammatory response and an important influx of neutrophils into the udder [1,5].

Initiation of the inflammation is accompanied by the production in milk of several molecules in the early stages of infection such as the neutrophil chemo-attractants CXCL8 and C5a and the proinflammatory cytokines IL-1ß, IL-6 and TNFα [6,7].

A contribution of Mammary Epithelial Cells (MEC) to the production of these different mediators has been suggested by different authors [8-11]. Indeed, the incubation of primary cell cultures of bovine Mammary Epithelial Cells (bMEC) obtained from healthy animals with bacteria, either *E. coli *or *S. aureus*, induces a strong response [11-13].

Recognition of bacteria by host cells, for example macrophages, dendritic cells or epithelial cells, relies upon so called Pattern Recognition Receptors (PRR) [14]. Such receptors belong to three different families namely the Toll-like (TLR), NOD-like (NLR) and RIG-1-like (RLR) receptors. Each of these receptors recognizes a set of bacterial motifs or Microbe Associated Molecular Patterns (MAMPs). For example, TLR4, TLR2 and TLR5 are respectively involved in sensing lipopolysaccharide (LPS), lipoteichoic acid (LTA) (and lipoproteins) and flagellin [14].

Homologues of human TLR receptors 1-10 have been identified in bovine and were shown to be expressed at different levels in the skin [15,16]. In the udder, the expression of TLR2, TLR4 and NOD2 has been demonstrated by several laboratories [17,18]. In agreement with the expression of these receptors, infusion of purified bacterial compounds recognized by these PRR lead to an inflammation of the udder mimicking the initial response to experimental infections with live bacteria. For example, infusion of LTA, muramyl-dipeptide (MDP) and LPS have already been shown to induce a neutrophil recruitment and to provoke symptoms similar, yet not identical, to those caused by live bacteria [1,18]. Most importantly, it seems that these bacterial agonists can act synergistically and increase the response of the udder [18].

Of these agonists that trigger inflammation of the udder, only LPS and MDP can be produced by *E. coli *strains and contribute to the inflammation observed during bovine mastitis. Yet, *E. coli *can also produce other agonists that can contribute to this inflammation by triggering TLR2 or other PRR such as TLR5 or NOD1. Actually, lipoproteins from the *E. coli *envelope can be detected by the TLR2 receptor in combination with either TLR1 (for tri-acylated lipoproteins) or TLR6 (for di-acylated lipoproteins) [14]. In addition, TLR5 was shown to recognize flagellin, the major constituent of flagella [19]. In human, NOD1 recognizes peptidoglycan fragments composed of gamma-D-glutamyl-meso-diaminopimelic acid (iE-DAP) or N-acetylmuramyl-L-alanyl-γ-D-glutamyl- meso-diaminopimelic acid (mur-TriDAP) [20].

Recognition of both flagellin and peptidoglycan fragments is an important issue given the role these MAMPs can play during an infection. Indeed, flagellin is a major player in the recognition of mucosal pathogens [19]. NOD1 has also been demonstrated as an important mediator in controlling infections by, for example, *Listeria monocytogenes, Helicobacter pylori *or entero-invasive *E. coli *[21-23]. Furthermore, it was recently shown that NOD1 could play a role in the maturation of the immune system and that its stimulation could modify the response of the host in a case of neutrophilic inflammation [24,25].

In order to better characterize how *E. coli *is recognized by bMEC, we investigated the repertoire of *E. coli *MAMPs that could be recognized by bMEC. MAMPs tested are known agonists of different innate immune receptors such as TLR2/TLR1 or TLR2/TLR6 heterodimers, TLR4, TLR5, NOD1 and NOD2.

## Materials and methods

### Reagents

C12-iE-DAP (C12-D-γ-Glu-mDAP), Tri-DAP (L-Ala-γ-D-Glu-mDAP), synthetic diacylated (Pam2CSK4) and triacylated (Pam3CSK4) lipopeptide, ultra pure LPS from *E. coli *K-12, bacterial flagellin from *S. enterica *serovar Typhimurium were obtained lyophilized from InvivoGen (Invivogen, France).

Agonists were made soluble in sterile pyrogen-free water and then diluted in cell culture medium.

### Experimentally induced mastitis

Healthy mid-lactating Holstein cows of the experimental herd of the Institute at Nouzilly were selected on the basis of an absence of detectable bacterial growth from two weekly consecutive aseptically collected milk samples in their four mammary quarters and less than 100 000 cells/mL in milk. The cows were in their second or third lactation and between 2 and 6 months in lactation; they were milked twice a day, at 0800 and 1600 h. The use and care of the cows in this study were approved by the Regional Committee of Ethics for Animal Experimentation (CREEA) (approval CL2007-47). At time zero, five cows received in the lumen of quarters through the teat canal either 0.5 mL RPMI 1640 medium (Gibco), C12-iE-DAP (10 μg), Tri-DAP (50 μg). The fourth quarter was left untreated. A second group of five cows received, at time zero, either 0.5 mL RPMI 1640 medium (Gibco) or purified flagellin (5, 1 or 0.1 μg). The infused quarters and the control quarter were aseptically sampled just before the morning milking, and infusion was carried out within 30 min post milking. The quarters were then sampled at 4, 8, 12, 24, 32, 48 and, in the case of NOD1 agonists, 72 h post infusion (hpi). Cells in milk were counted with an automated cell counter (Fossomatic model90; Foss Food Technology, Hillerod, Denmark) as described previously [26]. The occurrence of new infections in the quarters under experiment was verified by plating milk samples onto blood agar plates. Results from quarters infected during the experiment were discarded leading to the removal of results from one quarter in the control group of the each experiment, one quarter infused with C12-iE-DAP and one infused with flagellin (1 μg).

### Reverse transcription and qPCR analysis

Total RNA was extracted from bMEC by using the NucleoSpin RNA II extraction kit (Macherey-Nagel, Düren, Germany), and the residual genomic DNA was removed by using DNase digestion with RNase-free DNase (Macherey-Nagel). The total RNA quantity and quality were assessed by using a NanoDrop spectrophotometer (NanoDrop Technologies, Wilmington, DE, USA). Total RNA (1 μg) was then reverse transcribed to cDNA using random hexamers and SuperScript RT III (Invitrogen) according to the manufacturer's instructions. Diluted cDNA samples were stored at 4°C until use. Primers used in this study are listed in Table 1. Relative quantities of gene transcripts were measured as described previously [27].

**Table 1:** List of primers used in this study

Gene	Primer ID	Primer sequence	Size of amplicon	Annealing temperature (°C)
18S	18sF	CGGGGAGGTAGTGACGAAA	196 bp	62
	
	18sR	CCGCTCCCAAGATCCAACTA		

ACTB	ACTB f	ACGGGCAGGTCATCACCATC	166 bp	64
	
	ACTB r	AGCACCGTGTTGGCGTAGAG		

PPIA	PPIA Fq	TCCGGGATTTATGTGCCAGGG	206 bp	66
	
	PPIA Rq	GCTTGCCATCCAACCACTCAG		

TLR1	TLR1 Fq	ACCCTACTCTGAACCTCAAG	142 bp	62
	
	TLR1 Rq	GACTGCACACTGGATTTCTG		

TLR2	TLR2 Fq	ACTGGGTGGAGAACCTCATGGTCC	307 bp	62
	
	TLR2 Rq	ATCTTCCGCAGCTTACAGAAGC		

TLR4	TLR4 F2q	GCATGGAGCTGAATCTCTAC	238 bp	62
	
	TLR4 R2q	CAGGCTAAACTCTGGATAGG		

TLR5	TLR5 Fq	TTCCTGCAACCTCACCCAAG	192 bp	62
	
	TLR5 Rq	CTGAGATTGGGCAGGTTTCG		

TLR6	TLR6 Fq	CTCCGGGAGATAGTCACTTC	297 bp	62
	
	TLR6 Rq	GGCCCTGGATTCTATTATGG		

NOD1	NOD1 F2q	TGGTCACTCACATCCGAAAC	218 bp	62
	
	NOD1 F2q	AGGCCTGAGATCCACATAAG		

NOD2	NOD2-f	CCCAGGGGCTCAGAACTAACA	238 bp	62
	
	NOD2-r	CCTTCATCCTGGACGTGGTTC		

### Quantification of CXCL8 by ELISA

CXCL8 concentrations were measured in skim milk by ELISA as described [11,28]. The lowest limit of detection and level of quantification in milk were 0.01 and 0.03 ng/mL for CXCL8, respectively.

### Culture and stimulation of bMEC

bMEC were isolated from five lactating cows as previously described and cryopreserved in liquid nitrogen [10]. Cells were used at their third passage and cultivated as described [27]. Briefly, cells were seeded into 24-well tissue culture plates at a density of 10^5 ^cells/well and cultured until confluence in a growth medium made up of D-MEM-F12 advanced medium with 2 mM L-glutamine, 20 mM HEPES, 50 μg/mL IGF-1, 10 μg/mL FGF, 10 μg/mL EGF and 1 μg/mL hydrocortisone as additives. The growth medium was then replaced with stimulation medium made up of Advanced D-MEM-F12 medium with 2 mM L-glutamine, 20 mM HEPES, and 4 ng/mL hydrocortisone as additives. Stimulations with bacterial agonists were carried out 16-24 h later. The medium was removed, and agonists (uLPS, C12-iE-DAP, Pam2CSK4, Pam3CSK4, flagellin) were added at the desired concentration in 1 mL of stimulation medium. Control wells were treated with stimulation medium only. 5 h after exposure to the MAMP, culture medium was aspirated and stored at -20°C.

### Statistical analysis

Statistical analyses of the concentrations of CXCL8 or SCC in milk were performed with the nonparametric Friedman test. Comparisons of CXCL8 concentrations in bMEC supernatants were done using the Kruskal and Wallis test followed by multiple comparisons with a Bonferoni correction. A *P *value < 0.05 was considered significant.

## Results

### Multiple PRRs are expressed by bMEC

**Figure 1:**
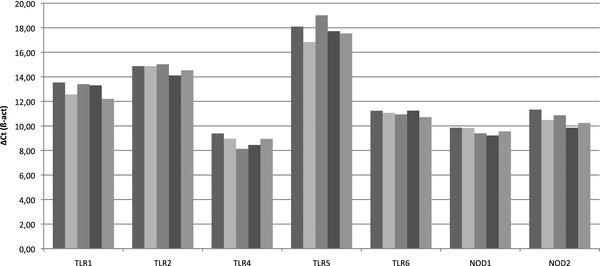
**Expression of PRR by bMEC**. Quantitative RT-PCR was performed on RNA obtained from bMEC isolated from 5 different cows. ΔCt values (Ct target--Ct for ß-actin gene) for each bMEC are represented on the graph. PCR efficiency was above 90% for all genes and Ct were obtained for an identical fluorescence threshold for all genes.

In a first attempt to characterize the repertoire of bacterial agonists recognized by the bovine udder, we investigated the expression of different Pattern Recognition Receptors (PRR) by RT-qPCR. PRR investigated are those homologous to mammalian receptors involved in recognition of LPS (TLR4), peptidoglycan (NOD1 and NOD2), lipoproteins (TLR2, TLR1 and TLR6) and flagellin (TLR5). Expression of all these PRRs was observed in the bMEC preparations isolated from the five different cows (Figure 1). Yet, expression of TLR5 was found to be much lower than that for other TLRs: indeed, although the PCR efficiencies were similar for all genes analyzed (above 90%), the ΔCt value for TLR5 was on average between 3 and 9 cycles above that of other genes. On the contrary, TLR4 and NOD1 were the most highly expressed receptors.

### bMEC respond to stimulation by LPS, peptidoglycan fragments but not flagellin

**Figure 2:**
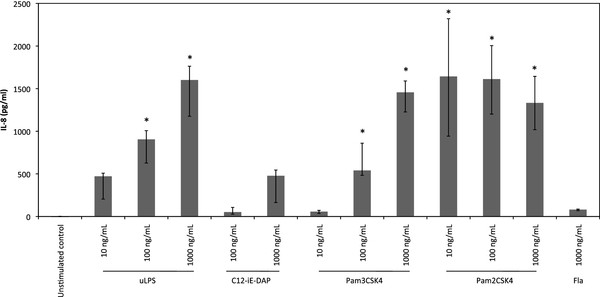
**CXCL8 secretion by bMEC after stimulation with different bacterial agonists**. bMEC from five different cows were seeded in 24-well plates at a concentration of 10^5 ^cells/well. Twenty-four hours prior to stimulation, the culture medium was replaced by stimulation medium. Cells were incubated in the presence of different purified agonists at the indicated concentrations. CXCL8 concentration in the supernatant was measured by ELISA 5 h after initiation of the stimulation. Data are median values and interquartile ranges. *statistical significance (*P *< 0.05) of values from stimulated versus unstimulated bMEC.

To investigate whether these transcription results translated into functional differences we investigated the response of bMEC to the different agonists recognized by these PRRs. Results obtained confirmed previous studies showing that bMEC respond to stimulation by agonists such as LPS, MDP or LTA by the production of CXCL8 (Figure 2 and data not shown) [18]. As all bMEC used in this study also express receptors for peptidoglycan fragments and lipoproteins, we investigated whether this was correlated to response to these compounds. Our results demonstrate that the NOD1 agonist C12-iE-DAP induces the production of CXCL8 in a dose dependent manner starting at 100 ng/mL C12-iE-DAP (Figure 2). bMEC also responded to Pam3CSK4 and Pam2CSK4, two lipoprotein analogs respectively agonists of the heterodimer receptors TLR1/2 and TLR2/6 (Figure 2). Interestingly, a strong response was observed with as little as 10 ng/mL of Pam2CSK4 and this response was not increased when using 100 ng/mL or 1000 ng/mL concentrations. Consistent with the low transcription of TLR5 by bMEC, these cells failed to respond to flagellin in a significant manner: indeed, even at a high concentration (1000 ng/mL), a very low production of CXCL8 was observed (Figure 2).

Altogether, these results indicate that, in addition to LPS, bMEC are able to sense and respond to TLR2, NOD1 and NOD 2 agonists but not to TLR5 agonists.

### The response of the udder to bacterial agonists mimics that of bMEC

Among the above tested agonists, Pam2CSK4 and Pam3CSK4 are supposed to act through stimulation of TLR2, a receptor that is known to be both expressed in vivo at the surface of mammary epithelial cells and functional as demonstrated by intramammary infusion of LTA [11,17]. We therefore focused our in ubero studies on receptors whose activation had never been studied previously in the cow.

**Figure 3:**
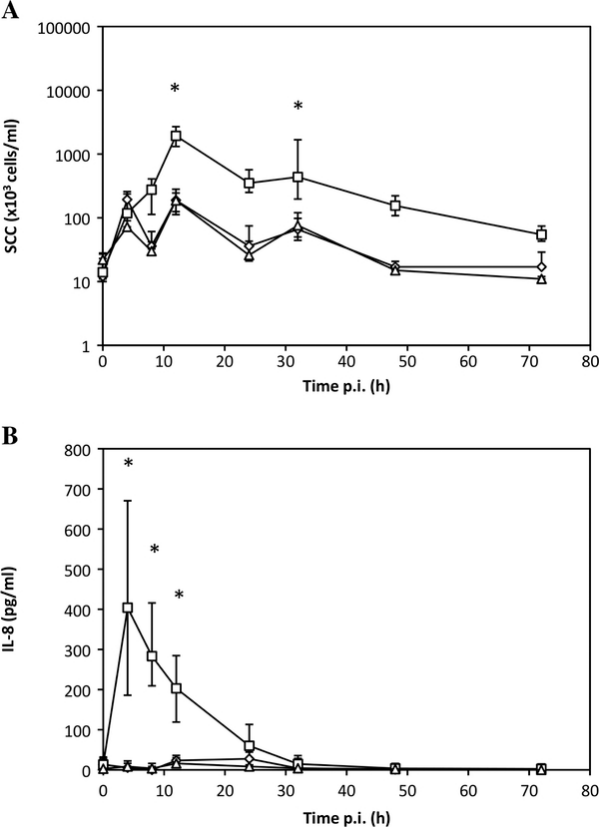
**A--Somatic cell counts (SCC) in quarters infused with C12-iE-DAP 10 μg (☐), Tri-DAP 50 μg (△) or control quarter (◊)**. B--CXCL8 concentration in milk from quarters infused with C12-iE-DAP 10 μg (☐), Tri-DAP 50 μg (△) or control quarter (◊). Purified agonists were infused into the udder of five different cows. SCC and CXCL8 were quantified in milk 4, 8, 12, 24, 32, 48 h post-infusion. Data are median values and interquartile ranges. *statistical significance (*P *< 0.05) of values before and after treatment.

We therefore tested the capacity of TLR5 and NOD1 agonists to induce inflammation when infused in the udder. C12-iE-DAP was infused in the mammary gland of 5 cows and we monitored the somatic cell count (Figure 3a) as well as the CXCL8 production in milk (Figure 3b). Our results clearly establish that this agonist induced cellular recruitment and CXCL8 production. Increased SCC compared to the control quarter was observed as early as 12 hpi. On the contrary, the use of another NOD1 agonist, Tri-DAP, failed to induce both a significant cellular recruitment and CXCL8 production in milk.

**Figure 4:**
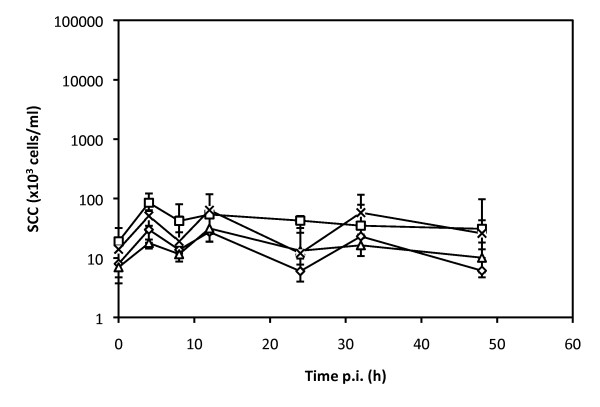
**Somatic cell counts (SCC) in quarters infused with flagellin 0.1 μg (☐), flagellin 1 μg (△); flagellin 5 μg (×) or control quarter (◊)**. Purified agonists were infused into the udder of five different cows. SCC and CXCL8 were quantified in milk 4, 8, 12, 24, 32, 48 h post-infusion. Data are median values and interquartile ranges. *statistical significance (*P *< 0.05) of values before and after treatment.

Flagellin was infused in the udder of 5 cows at three different doses, up to 5 μg: in all cases, no significant cellular recruitment was observed, consistent with the lack of TLR5 expression and response of bMEC to flagellin (Figure 4).

## Discussion

This study was undertaken in order to understand how *E. coli *can be sensed by the mammary gland and to better characterize the potential contribution of bMEC to the inflammation of the udder. The role of bMEC in initiating the inflammation has been suggested by several studies that showed that these cells were able to respond to different bacterial stimuli. In this study we investigated in more details the bacterial compounds that could be recognized by bMEC and whether these compounds were able to induce inflammation when infused into the udder. By a combination of transcription data, in vitro stimulation of bMEC and in ubero experiments we confirmed previous studies and showed for the first time that NOD1 could play a role in bovine mastitis while, on the contrary, TLR5 is not likely to be an important contributor to the inflammation of the udder.

More precisely, our results show for the first time that peptidoglycan fragments known to activate the NOD1 receptor in other mammals could be recognized by bMEC and are able to induce inflammation in ubero. This result is all the more important given the multiple activities that the NOD1 receptor is likely to play. Apart from a role in triggering the innate immune response, activation of NOD1 could also contribute to a more efficient reduction of the pathogen load by neutrophils as was shown in other infections [29]. Yet, we only observed inflammation of the udder with C12-iE-DAP and not with Tri-DAP. The major difference between these two agonists is that C12-iE-DAP is rendered membrane permeable by a lipophylic moiety which allows the molecule to better enter the host cell and activate NOD1 [30]. Indeed, the NOD1 receptor is cytoplasmic and its activation requires that the agonist is transported into the host cell [31].

The failure to induce inflammation of the udder with Tri-DAP is therefore likely to result from the lack of access of this agonist to the cytoplasm of host cells. Nevertheless, during the course of mastitis, it is possible that peptidoglycan fragments recognized by NOD1 can reach the cytoplasm of bovine cells following invasion of epithelial cells by *E. coli*. Indeed, invasion of mammary epithelial cells has been described for some strains of *E. coli *involved in recurrent mastitis cases [32]. Furthermore, expression of membrane transporters under particular circumstances like inflammation could transport peptidoglycan fragments, as was shown for MDP, a NOD2 agonist [18,33].

Most interestingly, we demonstrated that the TLR5 gene was only weakly transcribed and, consistently, that bacterial flagellin was not recognized by bMEC and did not induce inflammation of the udder. The failure of purified flagellin to induce inflammation of the udder was not expected since it is considered an important target of mucosal immunity [34]. Although the flagellin used in this study was purified from *S. enterica *serovar Typhimurium, it is known to activate most mammalian TLR5 receptors. Furthermore, the region of flagellin recognized by TLR5 is located in one of the two conserved regions of flagellin and is present both in *Salmonella *Typhimurium and *E. coli *flagellin [19,35]. It is therefore likely that, as for *Salmonella *Typhimurium flagellin, *E. coli *flagellin will not be detected by bMEC and does not contribute to the inflammation of the udder during *E. coli *mastitis.

Still, it is possible that TLR5 contributes to the response of the host when the infection worsens. In other pathologies, stimulation of TLR5 in epithelial cells is precluded by the basolateral localization of TLR5 and only occurs as a consequence of tissue damage whereby flagellin gets in contact with TLR5 receptors [36]. This situation is often the result of invasive pathogens and, in some instances, could occur in severe *E. coli *mastitis cases. One could also speculate that expression of TLR5 could be induced upon activation of other PRRs. Aside from detection by TLR5, flagellin, when translocated to the cytoplasm of the host cell, is also known to activate the inflammasome through stimulation of NLRC4 (also called IPAF) [37]. However, the contribution of this pathway to the response of the host to flagellin is still under debate [34]. The udder unresponsiveness to flagellin also suggests that milk macrophages were either too few or not reactive enough to set up inflammation.

Altogether, the above results support the idea that flagellin is not a critical motif for the early detection of *E. coli *in the healthy udder, neither through the activation of TLR5 nor that of NLRC4. Interestingly, proteomic analysis of the mastitis *E. coli *strain P4 have shown that expression of flagellin is decreased when bacteria are grown in milk compared to conventional laboratory growth medium [38].

Our results also show that bMEC are also able to sense the presence of lipoproteins through the TLR1/2 and TLR2/6 heterodimers. Both agonists used in this study induced a significant response of bMEC, Pam2CSK4 being more potent than Pam3CSK4 at inducing CXCL8 secretion by bMEC. Such results are not surprising since expression of the TLR2 receptor has already been shown in bMEC and was increased in infected udders [8,17]. In addition, infusion of the TLR2 agonist LTA in the udder of cows was shown to induce inflammation [18]. A new finding of this study is that bMEC reacted strongly to much lower concentrations of Pam2CSK4 than to Pam3CSK4. This higher reactivity is in line with the higher expression of TLR6 compared to TLR1 by bMEC. Nevertheless, although di-acyl lipoproteins can be found in *E. coli*, most *E. coli *lipoproteins are tri-acylated proteins [39]. It is therefore likely that the response of bMEC to Pam3CSK4 resembles most to that induced by *E. coli *lipoproteins.

Regarding the specificity of bovine PRRs, one should bear in mind that the specificity of these receptors might not be strictly identical to that of human/mouse PRRs, as reviewed by Werling et al. [40]. These authors actually state that bovine TLR1/TLR2 heterodimers could sense both Pam2CSK4 and Pam3CSK4. Concerning TLR5, studies with *Salmonella *Typhimurium flagellin indicated that it could activate bovine TLR5 expressing macrophages [41]. The specificities of bovine NOD1 and NOD2 receptors have not been characterized so far.

Investigations described in this manuscript were obtained with purified agonists used alone. Our recent results with MDP and LTA indicate that agonists can act synergistically. Because a bacterium expresses several MAMPs at a time such a synergy is likely to best mimic what bMEC are exposed to during *E. coli *mastitis. Synergy between MAMPs has already been observed in other models: in mice, for example, inoculation of LPS after an MDP treatment increases the immune response [42]. Synergy has also been observed with human monocytes and dendritic cells [43]. Our preliminary data indicate that such a synergy may exist in ubero between LPS and MDP (data not shown). The relevance of these potential synergies is important in that it is likely to improve the recognition of an udder pathogen and lead to an early response of the host, which impacts the outcome of infection.

A corollary would be to study how different *E. coli *strains stimulate each of these different PRRs. Because our results were obtained with purified generic compounds, they do not necessarily reflect the full diversity of *E. coli *MAMPs that can be recognized. Indeed, the *E. coli *species is very diverse: recent genomic studies have shown that of the 4500-5000 genes that one *E. coli *strain might contain only approx. 1800 genes are found in all *E. coli *strains [44]. Mastitis *E. coli *isolates has only been incompletely characterized. The presence of different genes characterized as virulence factors in other *E. coli *pathotypes has been investigated by several authors but none has been shown to be highly prevalent in mastitis *E. coli *strains. Recently, a preliminary study showed that the prototypical *E. coli *mastitis strain P4 contains several unique regions [45]. Although no potential virulence genes have been identified as specific of mastitis *E. coli *strains, the way *E. coli *stimulates the host cells might play a role in the inflammation of the udder.

In conclusion, bMEC are equipped with innate immunity receptors that allow them to sense several *E. coli *MAMPs. Overall, expression of PRRs as expressed by qPCR mirrored the reactivity of bMEC as assessed by the production of CXCL8, and the results obtained in bMEC in vitro were in good agreement with the in ubero studies. Such findings are in line with the concept that bMEC are key players in initiating neutrophil inflammation during *E. coli *mastitis. Recognition of several MAMPs at a time could contribute to the onset of an early response of the cow after infection by *E. coli*. These results pave the way to a better understanding of the early steps of the inflammation triggered by *E. coli *entrance into the udder. The precise contribution of the different PRR to this response is now necessary and deserves further studies.

## Competing interests

The authors declare that they have no competing interests.

## Authors' contributions

PRa and FBG conceived the study and participated in its design and coordination; PRa, FBG, AT and PC carried out collection and analysis of milk samples. AP, PG and PC performed and analyzed stimulation of bMEC and expression of PRR data. PG wrote the manuscript. AP, FBG, PRo and PRa helped writing the final version of the manuscript. All authors read and approved the final manuscript.
